# Antimicrobial Susceptibility among Colistin, Sulbactam, and Fosfomycin and a Synergism Study of Colistin in Combination with Sulbactam or Fosfomycin against Clinical Isolates of Carbapenem-Resistant* Acinetobacter baumannii*

**DOI:** 10.1155/2018/3893492

**Published:** 2018-01-18

**Authors:** Sombat Leelasupasri, Wichai Santimaleeworagun, Tossawan Jitwasinkul

**Affiliations:** ^1^Internal Medicine Unit, Phyathai II International Hospital, Bangkok, Thailand; ^2^Department of Pharmacy, Faculty of Pharmacy, Silpakorn University, Nakhon Pathom, Thailand; ^3^Antibiotic Optimization and Patient Care Project by Pharmaceutical Initiative for Resistant Bacteria and Infectious Diseases Working Group (PIRBIG), Silpakorn University, Nakhon Pathom, Thailand; ^4^Department of Biopharmacy, Faculty of Pharmacy, Silpakorn University, Nakhon Pathom, Thailand

## Abstract

This* in vitro* study aimed to determine the activity of colistin plus sulbactam and colistin plus fosfomycin against carbapenem-resistant* A. baumannii* (CRAB). Fifteen clinical isolates were obtained from patients admitted to Phyathai II International Hospital, Bangkok, Thailand, from August 2014 to April 2015. The antimicrobial susceptibilities of colistin, sulbactam, and fosfomycin were evaluated using the E-test or broth microdilution and the synergistic activity of the antibacterial combinations (colistin plus sulbactam or fosfomycin) was determined using the chequerboard method. Clonal relationships were explored using repetitive element palindromic- (REP-) PCR. The CRAB isolates were categorized by REP-PCR in 8 groups [A-H]. All CRAB isolates were universally susceptible to colistin but only 20.0% were susceptible to sulbactam. The MIC ranges for colistin, sulbactam, and fosfomycin were 0.75–2 mg/L, 2–96 mg/L, and 64–256 mg/L, respectively. A chequerboard assay revealed that the rates of synergistic and additive effect rates of colistin plus sulbactam and colistin plus fosfomycin were 53.3% and 73.3% of isolates, respectively. No antagonistic effect in any colistin-based combination was observed. However, almost CRAB strains in clone A showed the synergy or additive effects of colistin-sulbactam combination, whereas the other clone (B-H) mostly showed indifferent effects. In conclusion, colistin plus sulbactam and colistin plus fosfomycin against CRAB seem to be interesting option but the efficacy in clinical use has to be evaluated.

## 1. Introduction

Carbapenem-resistant* A. baumannii* (CRAB) is the most important pathogen in nosocomial infections worldwide causing high morbidity and mortality and increasing medical expenditure [1, 2]. Data from the National Antimicrobial Resistant Surveillance, Thailand, in 2015, showed that* A. baumannii* was ranked the third and second of pathogens isolated from all specimens and sputum, respectively. Considering the various CRAB strains, the report showed that* A. baumannii* was up to 70% resistant to imipenem or meropenem (National Antimicrobial Resistant Surveillance, Thailand, 2015); thus, CRAB infections are difficult to treat in an increasingly antimicrobial resistant era. One of the various strategies to improve clinical outcomes and to prevent emerging resistance during treatment is antimicrobial combination therapy [3, 4].

To date, colistin (polymyxin E) or polymyxin B-based combinations are recommended as treatments for CRAB because almost all strains of CRAB remain sensitive to polymyxins [5], while sulbactam and fosfomycin have better pharmacokinetic profiles with moderate and low protein binding. Sulbactam and fosfomycin are currently used at maximum dosage to cover some resistant strains [6, 7]. Certain studies have focused on determining the synergist effect of colistin plus sulbactam and colistin plus fosfomycin against* A. baumannii*. Those previous studies of* in vitro* synergism showed that colistin plus sulbactam might be a combined therapeutic option for CRAB treatment [3]. Moreover, colistin combined with fosfomycin revealed a synergistic effect or additive effects against CRAB strains or extensively drug-resistant* A. baumannii, *but only two studies addressed the colistin-fosfomycin combination with discordant results [8, 9].

As previously described, owing to the inconsistent findings of colistin-based combinations due to the variety of bacterial strains used in related studies from university medical schools, it is hard to reflect on or to generalize the beneficial results of such synergistic effects. We, therefore, performed this study to determine the* in vitro* synergistic effects of colistin plus sulbactam and colistin plus fosfomycin against clinical strains of CRAB.

## 2. Materials and Methods

### 2.1. Bacterial Strains

Clinical CRAB strains were isolated from inpatients admitted to Phyathai II International Hospital, a 550-bed private hospital for inpatient cases in Bangkok, Thailand, from August 2014 to April 2015. CRAB was defined as resistance to either imipenem, meropenem, or doripenem according to the Clinical and Laboratory Standards Institute screening criteria of carbapenem-resistant strains [10]. All clinical isolates of CRAB were firstly obtained from various specimens in each patient. All CRAB strains were kept at −20°C until tested. The institutional review board approved the research protocol with a waiver for informed consent [number ID0013/59].

### 2.2. Clonal Relationships

The clonal relationships of CRAB were evaluated using the REP-PCR method. The PCR reaction mixture had been described elsewhere [11]. Briefly, a couple primer (REP-forward: 5′-IIIGCGCCGICATCAGGC-3′ and REP-reverse: 5′-ACGTCTTATCAGGCCTAC-3′) was used to amplify the REP region under the following conditions, starting with heating at 94°C for 10 minutes, followed by 30 cycles of 94°C for 1 minute, 45°C for 1 minute, 72°C for 2 minutes, and finally 72°C for 16 minutes. The REP-PCR products were performed using agarose gel electrophoresis and were stained with ethidium bromide. The criterion for classifying the different clones was a pattern that differed from the at least three bands or more of REP-PCR [12].

### 2.3. Determination of Minimum Inhibitory Concentration

The MICs of sulbactam and fosfomycin were determined by the Epsilometer test (E-test; Liochem) method using Mueller-Hinton agar (Difco, Detroit, MI) plates, specifically, the E-test of fosfomycin supplemented with glucose-6-phosphate (G-6-P). The MICs of colistin were determined by broth microdilution using Mueller-Hinton broth II (Difco). The lowest concentration of an antimicrobial agent that inhibited visible growth of the organism was defined as the MIC. The quality controlled strain constituted* E. coli *ATCC 25922 [Department of Medical Sciences Type (DMST) culture collection, Bangkok, Thailand], which was used to monitor the accuracy of MIC determination based on CLSI 2017.

The MIC findings of sulbactam, colistin, and fosfomycin were interpreted using standard breakpoint criteria. Colistin and sulbactam susceptible breakpoints were defined as ≤2 mg*/*L and ≤4 mg*/*L, respectively, according to CLSI 2017. Due to the lack of a standard MIC breakpoint for fosfomycin against* A. baumannii *in CLSI, an MIC resistance breakpoint of ≤32 mg*/*L was established for fosfomycin according to the European Committee on Antimicrobial Susceptibility Testing 2017 (EUCAST).

### 2.4. Synergistic Testing

The synergistic effects of antimicrobial combinations (colistin plus sulbactam and colistin plus fosfomycin) were determined by the chequerboard technique. This technique was performed with Mueller-Hinton broth II (Difco), supplemented with 25 *μ*g/mL G-6-P in a fosfomycin combination. All samples were incubated at 37°C for 18 hours. The fractional inhibitory concentration indices (FICI) were calculated for each of the paired antibiotics and were interpreted as ≤0.5 = synergistic, 0.5–≤1.0 = additive effect, >1–4 = no interaction, and ≥4 antagonistic effect.

## 3. Results

Fifteen CRAB isolates were obtained from various sites. No particular strains were isolated repeatedly within the same patient (Table 1). According to the clonal relationship study, the 15 samples could be classified into 8 clones including A-H. Most strains were in clone A (*n* = 8), whereas the remaining 7 strains were in each member of B-H.

Among 15 CRAB isolates, the MIC ranges for colistin, sulbactam, and fosfomycin were 0.75–2 mg*/*L, 2–96 mg*/*L, and 64–256 mg*/*L, respectively. The percentage of susceptible strains was 100 and 20 for colistin and sulbactam, respectively, according to CLSI breakpoint, but all CRAB strains showed resistance to fosfomycin according to EUCAST breakpoint.

The antibiotic combination findings from the chequerboard study are shown in Table 2. Both synergistic and additive effects were found for colistin plus sulbactam in 2 of 15 isolates (13.3%) and 6 of 15 isolates (40.0%), respectively. Both synergistic and additive effects were also seen for the colistin-fosfomycin combination in 4 of 15 isolates (26.7%) and 7 of 15 isolates (46.7%), respectively. Indifferent effect was seen for colistin-sulbactam and colistin-fosfomycin combination in 7 of 15 isolates (46.7%) and 4 of 15 isolates (26.7%), respectively. Likewise, no antagonistic effects were observed with any of the combinations of antimicrobial agents studied.

## 4. Discussion

According to these results, all CRAB isolates were universally susceptible to colistin. Our findings were similar to those of several related studies in Thailand, indicating that colistin remained the most chosen therapeutic choice for CRAB infections [8, 13, 14]. While almost all of the CRAB strains were resistant to sulbactam and fosfomycin with quite high MICs, 86.7% of the CRAB isolates had a sulbactam MIC ≤32 mg/L. Such an MIC threshold could be dealt with using the clinically maximum daily dosage of 12 gm of sulbactam [15]. This 12-gm daily regimen also evaluated the efficacy and safety of MDR* A. baumannii* VAP [7]. Thus, the high dose of sulbactam seemed to be an alternative option for CRAB infections as long as the pathogens have MICs up to 32 mg/L.

Unlike sulbactam, fosfomycin was reportedly used as a high dose regimen of 24 g/day, and a pharmacokinetic study of 8 g-fosfomycin intravenously every 8 h at a steady rate showed the percentage of time above MIC being only 61% for MICs of 32 mg/L, respectively. According to the MIC of our CRAB strains with more than 64 mg/L, fosfomycin was unacceptable monotherapy due to intrinsic resistance, but the role of fosfomycin combinations might be used for CRAB infections.

According to the present findings, the MICs of colistin plus sulbactam demonstrated in synergy testing ΣFICI ≤ 1 (synergistic effect plus additive effect) by 53.3%. Our results were similar to Pongpech et al. and Çikman et al. whose studies reported 53.3% and 45.5% synergy plus additive rates of colistin-sulbactam against MDR* A. baumannii,* respectively [13, 16]. Moreover, Santimaleeworagun et al. showed synergy plus additive rates of colistin-sulbactam combination against CRAB as high as 75% [8]. Contrarily, Thamlikitkul and Tiengrim reported only an 18.2% synergy plus additive rate with such a combination [14].

The colistin plus fosfomycin combination synergy was controversial. The colistin-fosfomycin combination in the present study and the synergy plus additive rates rather differed from the results from Santimaleeworagun et al. that showed 73.3% and 37.5%, respectively [8], but Wei et al. showed a synergistic effect of colistin-fosfomycin in one half of their tested isolates [9]. With only two reports on the colistin-fosfomycin combination, this antimicrobial combination was further studied to prove the benefit of colistin-fosfomycin against MDR* A. baumannii*.

As previously mentioned, a discordance of synergism had been found. Several explanations include various synergistic testing methods (such as the chequerboard test, E-test, or time-kill methods) and the different resistance mechanisms for testing* A. baumannii* in each* in vitro* synergy study. On the effect of different methods of synergy test, Sesli Cetin et al. found that the detection rate of synergy plus additive effect of polymyxin with sulbactam among the same sample of* A. baumannii* strains significantly differed between the chequerboard (5%) and the E-test methods (35%) [17].

Regarding the clonal relationships in this study, clone A (53.3%) was predominant. Almost CRAB strains in this group showed the synergy or additive effect of colistin plus sulbactam, whereas the other REP-PCR groups (B-H) mostly showed indifferent effect of a couple of antimicrobials. Moreover, certain strains showed varied synergy findings between colistin-sulbactam and colistin-fosfomycin. These different effects of individual clone might be from diversity of resistance mechanisms. Leite et al. found that all* A. baumannii* isolates harboring* bla*OXA-23-like, the combination of colistin-vancomycin, showed synergy but of the strains harboring* bla*OXA-143-like, only 40% presented synergism with the colistin-vancomycin combination [18].

Although the synergistic effect of* in vitro* study seems to be the therapeutic choices, the recent meta-analysis of clinical study found that clinical response, death, length of hospitalization, or toxicity, except microbiological response, did not change significantly between colistin-based combination therapy and colistin monotherapy for* A. baumannii* infection [19]. Thus, additional studies are needed to confirm the benefit of the colistin combination in CRAB infection.

Moreover, we performed only chequerboard testing, but this is the most widely used method [3]. However, synergy testing such as time-kill studies or E-test methods should be performed.

## 5. Conclusion

Colistin plus sulbactam and colistin plus fosfomycin showed synergistic or additive effect against some CRAB isolates. However, due to the high MIC of sulbactam or fosfomycin, the maximum dosage was considered in clinical practice. Further studies with a larger sample and more synergy methods to evaluate the* in vitro* activity are required.

## Figures and Tables

**Table 1:** Minimum inhibitory concentration (MIC) determination by E-test and susceptibility interpretation of colistin, sulbactam, and fosfomycin against carbapenem-resistant *A. baumannii* isolates.

Strains	Specimen	REP-PCR group	MIC (mg/L) determination		
			Colistin (interpretation)^a^	Sulbactam (interpretation)^b^	Fosfomycin (interpretation)^c^
AB 1	Blood	A	2 (S)	96 (R)	192 (R)
AB 2	Pus	A	1.5 (S)	4 (S)	192 (R)
AB 3	Blood	B	2 (S)	24 (R)	128 (R)
AB 4	Femur tissue	A	1.5 (S)	24 (R)	96 (R)
AB 5	Sputum	C	2 (S)	2 (S)	64 (R)
AB 6	Sputum	A	1.5 (S)	6 (S)	128 (R)
AB 7	Sputum	A	1.5 (S)	8 (I)	256 (R)
AB 8	Sputum	D	1.5 (S)	8 (I)	96 (R)
AB 9	Sputum	E	1 (S)	12 (I)	96 (R)
AB 10	Sputum	F	0.75 (S)	16 (R)	192 (R)
AB 11	Sputum	G	1 (S)	16 (R)	256 (R)
AB 12	Pus	H	1.5 (S)	96 (R)	256 (R)
AB 13	Sputum	A	1.5 (S)	12 (I)	192 (R)
AB 14	Sputum	A	1.5 (S)	8 (I)	128 (R)
AB 15	Urine	A	1.5 (S)	16 (R)	128 (R)

Breakpoint criteria as follows: ^a^colistin: susceptible; S, ≤2 mg/L; resistant; R, >4 mg/L, ^b^sulbactam: susceptible; S, ≤4 mg/L; intermediate; I, 8 mg/L; resistant; R, ≥16 mg/L and ^c^fosfomycin: susceptible; S, ≤32 mg/L; resistant; R, >32 mg/L.

**Table 2:** Antimicrobial combination test against carbapenem-resistant *A. baumannii* clinical isolates using chequerboard technique.

Strains	REP-PCR group	Colistin + sulbactam				Colistin + fosfomycin			
		FICI (Col)	FICI (Sul)	ΣFICI (Col + Sul)	Interpretation (Col + Sul)	FICI (Col)	FICI (Fos)	ΣFICI (Col + Fos)	Interpretation (Col + Fos)
AB 1	A	0.06	2	2.06	Indifference	0.5	0.5	1	Additive
AB 2	A	0.25	0.5	0.75	Additive	0.5	0.13	0.63	Additive
AB 4	A	0.13	0.25	0.38	Synergism	0.5	0.25	0.75	Additive
AB 6	A	0.5	0.06	0.56	Additive	0.5	0.25	0.75	Additive
AB 7	A	0.25	0.25	0.5	Synergism	0.25	0.13	0.38	Synergism
AB 13	A	0.13	0.5	0.63	Additive	0.13	1	1.13	Indifference
AB 14	A	0.5	0.5	1	Additive	0.5	0.5	1	Additive
AB 15	A	0.03	1	1.03	Indifference	0.13	0.25	0.38	Synergism
AB 3	B	0.5	0.5	1	Additive	1	0.25	1.25	Indifference
AB 5	C	0.13	1.0	1.13	Indifference	0.25	0.25	0.5	Synergism
AB 8	D	0.06	1	1.06	Indifference	0.25	0.5	0.75	Additive
AB 9	E	0.008	1	1.01	Indifference	0.03	0.25	0.28	Synergism
AB 10	F	0.004	2	2.00	Indifference	0.02	1	1.02	Indifference
AB 11	G	1	0.004	1.004	Indifference	0.01	1	1.01	Indifference
AB 12	H	0.06	0.5	0.56	Additive	0.25	0.5	0.75	Additive

The fractional inhibitory concentration indices (FICI) were interpreted as ≤0.5 = synergistic, >0.5–<1.0 = additive effect, >1–4 = indifference, and ≥4 antagonistic effect.
